# Health Equity in National Cancer Control Plans: An Analysis of the Ontario Cancer Plan

**DOI:** 10.15171/ijhpm.2019.40

**Published:** 2019-06-16

**Authors:** Ambreen Sayani

**Affiliations:** ^1^School of Health Policy and Management, Faculty of Health, York University, Toronto, ON, Canada.; ^2^MAP Centre for Urban Health Solutions, Li Ka Shing Knowledge Institute, St. Michael’s Hospital, Toronto, ON, Canada.

**Keywords:** Cancer Control Plan, Health Equity, Ontario

## Abstract

**Background:** National cancer control plans (NCCPs) are important documents that guide strategic priorities in cancer care and plan for the appropriate allocation of resources based on the social, geographic and economic needs of a population. Despite the emphasis on health equity by the World Health Organization (WHO), few NCCPs have a focus on health equity. The Ontario Cancer Plan (OCP) IV, (2015 to 2019) is an example of an NCCP with clearly defined health equity goals and objectives.

**Methods:** This paper presents a directed-content analysis of the OCP IV health equity goals and objectives, in light of the synergies of oppression analytical framework. Results: The OCP IV confines equity to an issue of access-to-care. As a result, it calls for training, funding, and social support services to increase accessibility for high-risk population groups. However, equity has a broader definition. And as such, it also implies that systematic differences in health outcomes between social groups should be minimal. This is particularly significant given that socially disadvantaged cancer patients in Ontario have distinctly poorer cancer-related health outcomes.

**Conclusion:** Health systems are seeking ways to reduce the health equity gap. However, to reduce health inequities which are socially-based will require a recognition of the living and working conditions of patients which influence risk, mortality and survival. NCCPs represent a way to politically advocate for the determinants of health which profoundly influence cancer risk, outcomes and mortality.

## Key Terms

**Health equity:** The term health equity is used to define differences in health and access to healthcare amongst individuals who have different levels of underlying social advantage or disadvantage.^1^

**Social determinants of health (SDH):** The SDH are the conditions in which we are born, grow, work and age. These conditions shape our daily lives, and impact our opportunities for health. They are themselves determined by the social and economic policies of the jurisdictions in which we live.^2^

**Social location:** The social location is also referred to as the social position. The rank order an individual occupies on a hierarchical ladder of power, privilege and prestige is called the social location. Many elements work in tandem, or intersect to influence the social location. These include material and social resources such as wealth and income; gender, age, religious affiliation, and mental or physical disability.^3^

**Social inequity:** Social inequity is a term which is used to describe the unequal distribution of power, privilege and prestige across a society. Inherently, individuals who occupy positions of social advantage by virtue of their personal wealth and credentials are more able to access resources and services^4^ thereby creating further differentiation between social groups.

**Social structural inequality:** Social structural inequality refers to the hierarchical ordering of people based on their position in society that is determined by their level of power, prestige and privilege. When social inequality becomes systematically entrenched in a society such that it is institutionalised into policies and procedures that continue to differentiate between social groups, it is called social structural inequality or social stratification.^5^

**Cancer care continuum:** The cancer care continuum is a conceptual pathway that represents the journey of an individual right through from cancer prevention, to screening, treatment, survivorship and end-of-life care.^6^

## Introduction

Since the year 2000, there has been a steady increase in the number of national cancer control plans (NCCPs) across the globe.^7^ According to the World Health Organization (WHO), NCCPs are important tools to reduce incidence and mortality due to cancer, as well as improve the quality of life for cancer patients and their families.^8^ Ideally, systematic and evidence-based goals are set by NCCPs based on the demographic, social and resource needs of the local population; with subsequent action on these goals leading to improvements in cancer prevention, early diagnosis, treatment and mortality.^8^ As such, NCCPs are an important bridge between population needs and population health objectives and serve as a strategic roadmap for interventions and policy design.^8^ Whilst the WHO clearly highlights the importance of ensuring equity in the cancer-related health outcomes of a population,^8^ recent evaluations of 527 NCCPs across the globe have revealed that only a third of the plans have a focus on health equity.^7^ Furthermore, it has been pointed out, that equity objectives in NCCPs typically fail to contextualise exactly how health equity will be achieved and/or measured.^9^

The cancer care system in Ontario, Canada is monitored and managed by an organization called Cancer Care Ontario (CCO). Every 4 years CCO releases a policy document called the Ontario Cancer Plan (OCP) to guide resource allocation and planning. The most recent OCP, OCP IV (2015 to 2019) has a clearly defined health equity goal with an ambition to “ensure health equity for all Ontarians across the cancer system.”^6^ In addition, the OCP IV distinctly articulates its strategic health equity objectives by committing to work with Indigenous populations, collect data, and inform locoregional policies and programs.^6^

Given that the OCP IV is amongst a handful of NCCPs that have a health equity priority, analysing the OCP IV from a health equity lens presents some novel opportunities. For starters, this plan can be considered an exemplar for how other health systems may choose to categorise their equity goals. It is therefore important to shed light on the health equity discourse in the OCP IV. Furthermore, since the document is a strategic roadmap, it is meaningful to understand the health equity implications of the planned objectives and the ability of proposed interventions to respond to the unequal allocation of the determinants of health which are socially based.

## Background

CCO acts as a provincial advisor to the government and directly supervises approximately 1.8 billion dollars of cancer care through hospitals and service providers.^10^ CCO coordinates priorities and targets for cancer delivery between CCO, Regional Cancer Programs and the Ministry of Health and Long-Term Care, through a strategy document called the OCP. As an advisory document to the government, the OCP identifies unmet needs of cancer patients and their families, and brings to the limelight actionable methods to address them. As a result, the OCP-set, and government-endorsed goals guide the allocation of funds, resources and health workers across the province to enhance the cancer care system.

The OCP I was released in November 2004 and was a three-year roadmap that guided cancer care from 2005 to 2008. Along with the second OCP, OCP II (2008 to 2011), these two strategic documents were focused on enhancing the capacity of the cancer system to keep up with the increasing incidence of cancer. They also laid the groundwork for quality monitoring and evaluation by synthesizing data collection into key performance indicators. The OCP III (2011 to 2015) was a 4-year plan committed to quality improvements across the cancer care continuum and a person-centered approach to care. The OCP IV (2015 to 2019) is the latest strategic plan from CCO that aims to further enhance the patient’s quality of life across the cancer care continuum in a manner that is both safe and equitable.^6^

Oversight for the work done by CCO is carried out by the Cancer Quality Council of Ontario, a partially independent body that evaluates the work carried out by the CCO and reports back to the CCO-Board and Ministry of Health and Long-Term Care. The Cancer Quality Council of Ontario also releases public reports thereby creating a degree of transparency into the functioning of Ontario’s cancer system.^11^ Recent reports have rated CCO’s biomedical aspects of cancer care, such as safety, effectiveness and accessibility as “good”; whereas more holistic measures of patient care, such as responsiveness, equity and integration demonstrate room for improvement.^11^ These findings are in line with the Auditor General of Ontario’s report, which found inequities in cancer care across the province.^12^

In Ontario, innovative ways to tackle cancer are becoming increasingly important, and this is reflected in current policies and programs which strive to promote prevention, reduce risk, and make health services more accessible. What has not been well reflected in public policy or national strategy however, is the growing equity gap between those who survive cancer and those who do not. Almost twenty years ago, Mackillop et al,^13^ studied 360 000 cases of invasive cancer in Ontario and demonstrated how individuals from lower income groups were not only more likely to die of cancer, but that they were also more likely to die sooner than their richer counterparts. Interestingly, Booth et al,^14^ highlighted similar findings in a study conducted almost a decade later. Apparently, the more money one has, the greater their odds of overcoming cancer. And frighteningly, this is a finding that has persisted over time.

The SDH such as income and education are known to correlate with the stage of diagnosis,^14^ survival from,^14^ and quality of life^15^ with cancer.^16^ Recent austerity measures in Canada, with a retrenchment of the state from the provision of social services is increasing the pool of socially disadvantaged people.^17^ As a result, poverty levels, homelessness, food insecurity, and precarious working conditions are on the rise with a subsequent increase in income inequality.^17^ Whereas the correlation between income inequality and health inequality is well documented,^18^ this association is less apparent when it comes to the planning and delivery of health services for cancer care.

Typically health systems have a focus on the acute management of illness, and for cancer systems this means that resources are frequently allocated to the delivery of good quality care for prevalent cancers. Populations that are harder to reach, or have poorer cancer-related health outcomes can be defined as priority populations,^19^ and for health systems an objective has been to enhance access to care via increased investment in material and human resources to deliver care. An inherent assumption in this process however, is that the accessibility of care will lead to its utilization, whereas evidence clearly points to the unequal utilization of health services amongst population groups based on their social location particularly for cancer care.^20^ Indeed, the structure and design of health services can impose a profound barrier to socially disadvantaged populations who may end up systematically excluded from programs, resulting in a worsening of health inequities.^21^

This paper explores the equity goal of the OCP IV: 2015-2019 in light of the “synergies of oppression” framework constructed by McGibbon and McPherson,^22^ and highlights how a narrow conceptualisation of the term “equity” can be interpreted as top-down biomedical interventions which inherently favour the socially advantaged over those that are not. An implication of this is that social structural inequalities are almost completely ignored, and may have a counter productive effect of increasing the equity gap between rich and poor cancer patients.

## Methods

This study is a directed content analysis^23^ of the OCP IV (2015 to 2019) equity goal conducted using the synergies of oppression analytical framework^22^ and guided by the multilevel cancer systems model described by Taplin et al.^24^

McGibbon and McPherson^22^ use an intersectional lens to link feminist intersectionality theory with feminist political economy and complexity theory, to construct the “synergies of oppression” framework which illuminates how (*i*) social identities such as gender, race, class, disability, ethnicity, migrant status, culture and sexual orientation; (*ii*) SDH such as education, income, job, housing and food security; and (*iii*) social geography such as rural location, service accessibility, cultural segregation, environmental toxins, etc; can all cross paths and intersect to varying degrees resulting in lost opportunities for the health and wellbeing of women. Although the synergies of oppression framework has been used to illuminate gender specific inequalities,^22^ the concept is widely applicable to understand the role of social structural inequalities in mediating health inequities. In this paper, this analytical lens has been used as a tool to contextualize inequities in cancer care and outcomes.

Taplin et al^24^ describe a multilevel ecological model of influencers across the cancer care continuum, based on the six dimensions of quality as described by the Institute of Medicine: safety, effectiveness, patient-centredness, equity, efficiency and timely care.^25^ Taplin et al^24^ define patient-level success across the cancer care continuum as changes in patient risk, stage of diagnosis, quality of life, quality of death and financial burden; with these indicators subsequently feeding into long term markers of population-level success such as reduced cancer-related mortality and morbidity. The framework provided by Taplin et al^24^ was used to develop the methodological line of inquiry and to develop the study questions.

The study questions were:

1. How does the OCP IV (hereafter termed OCP) conceptualise the term equity?
2. When planning for risk reduction, does the OCP consider social inequalities as well as recognized biological factors?
3. What are the socioeconomic implications of cancer treatment, and how does this influence equitable outcomes of care?
4. How does the OCP propose to make end-of-life care choices equitable, and is the relationship between care choices and social location reflected in the OCP?

## Results

### Concepts of Equity in the Ontario Cancer Plan

After a call to action from the WHO commission to close the equity gap both between and within nations,^2^ health policies are increasingly building equity into their population plans. However, it is important to contextualize the scope and breadth with which health policies use the term equity. How equity is defined and understood has direct implications into the translation of the policy words into actionable plans. For example, some policies specifically use the term equity in relation to geographic and financial accessibility of healthcare; whilst others use it to highlight differences in health status amongst groups.^26^

When strategizing equity related goals, the OCP mentions the following policy objectives:

“*Develop locally relevant policies and programs in partnership with community service providers to improve access to services for specific populations and support healthcare providers with training, data and tools to deliver equitable services.”^6^*

*“Advise governments in the development of provincial policies and programs to improve access to services for specific populations, including equitable access to specialized services.”* ^6^

“*Ensure equitable access to palliative care for all Ontarians, including vulnerable populations.”^6^*

The OCP specifically links equity with accessibility. Hence the plan calls for: more centers, improved community outreach programs, enhanced provider cultural-linguistic competence, and identification of high-risk groups to facilitate access to care.

The OCP avoids planning for any health inequities that are beyond the scope of top-down biomedical interventions. Interestingly, the plan draws the link between equity and social identities only once in the entire plan. However, here too it is exclusively in the context of access:

*“Ontario’s population is diverse and geographically dispersed. Patient’s access to care and their health outcomes should not depend on demographic characteristics or where they live. Yet some Ontarians face significant, and often multiple, barriers in finding and accessing cancer services based on geography, race, culture, gender, age, sexual orientation, immigration status, and education.”* ^6^

The link between health equity, the SDH and barriers to care are also drawn only once in the OCP. Here too, it is in relation to accessibility of service:

*“We need to better understand the barriers that contribute to health disparities across the cancer care continuum, including barriers between health and community services to address SDH. We need to raise awareness among traditionally underserved populations about what services are available, how to access them and why it is important to do so.”* ^6^

### Risk Reduction 

In the cancer care community, modifiable risk factors for cancer have been identified as smoking, obesity, lack of physical activity, unhealthy diet, alcohol consumption, and occupational and environmental exposures.^27^

As a result, the OCP quantifies the number of preventable cancers, and hence the importance of action to reduce risk:

*“In high-income countries similar to Canada, an estimated 40% to 50% of cancers are associated with behavioural, occupational and environmental risk factors, and could be prevented. In light of our growing and aging population, initiatives that target modifiable risk factors take on added importance.”* ^6^

The OCP calls for reducing risk factors by identifying high risk individuals and community risk profiling to target interventions.

*“All Regional Cancer Centres developed programs to screen patients for smoking status and refer smokers to smoking cessation programs.”* ^6^

It is now well established that patterns of social disadvantage such as adverse working and living conditions are linked to the adoption of unhealthy lifestyle behaviours.^28^ Unfortunately, a failure to acknowledge this correlation in the OCP means that the plan falls short of fostering long term health equity in the population.

The OCP tasks individuals with the identification and management of their own cancer risk, and as such it suggests that logging onto an online portal can help reduce cancer risk:

*“MyCancerIQ launched in February 2015. This online cancer risk assessment tool will help to motivate Ontarians to reduce their risk of developing cancer and increase their participation in cancer screening.”* ^6^

It is important to understand however that this online tool may only be accessible, usable and beneficial to a select group of the population, most likely the socially advantaged. Biological risk can be shaped and structured by adverse working and living conditions which may not be amenable to individual choice. Examples of these include working night shifts,^29^ exposure to environmental toxins,^29^ and food insecurity^30^; all of which can contribute to a higher incidence of cancer. An online tool does not recognise social structural inequality which is a determinant of elevated cancer risk, and may inadvertently increase the health equity gaps based on participation by different population groups.

### Cancer Related Expenses and Implications for Equitable Care 

In Canada, 70% of health services are funded through the state.^31^ Of the remaining 30 percent, approximately half are covered through employment-sponsored health plans, and the other half are paid directly out-of-pocket by cancer patients or their families.^32^ Studies demonstrate how low income, lack of health insurance and high levels of co-payment are associated with lack of adherence to oral anti-cancer drugs^32^ which is crucial for long-term disease-free survival.^33^ In recognition of this the OCP mentions two new drug reimbursement programs:

*“Cancer Care Ontario’s Provincial Drug Reimbursement Program launched 2 new programs to improve access to necessary cancer treatments across the province: The Evidence Building Program facilitated funding for treatment to 345 patients while collecting real-world data on each medications clinical and cost-effectiveness; and the Case-by-Case Review Program helped approximately 50 cancer patients with immediately life-threatening circumstances receive treatments with drugs that would otherwise be unfunded.”* ^6^

Innovations in cancer therapy mean that intravenous chemotherapy is now increasingly available in an oral form.^34^ As a result, therapy is increasingly being delivered at home rather than in the hospital. This phenomenon is referenced in the OCP in relation to patient safety:

*“Patients and providers will be partners in designing how chemotherapy is delivered safely in the home.”* ^6^

What has not been mentioned in the OCP however, are the financial implications of changing the location of cancer therapy from in-hospital to at-home such that the responsibility to fund drugs is pushed from the state to the individual. The drug reimbursement plans mentioned in the OCP are not a response to the new ways in which cancer therapy is delivered or financed. Instead, the drug reimbursement plans serve select groups of patients, such as those on specific drugs and those in critical life-threatening situations.

On average cancer patients participate approximately 36% less in the labour market, and as a result lose about 26% of their income.^35^ In this context, out of pocket expenses can become increasingly significant as personal income may continue to decline. Indeed, one in six cancer patients in Ontario feel that out of pocket costs are “significant” and “unmanageable.”^32^ Circumstances such as low levels of workplace flexibility are associated with increased job-loss, and a greater delay in return to work.^36^ In addition, caregivers too may lose approximately 25% of their income^35^ due to the increased burden of care. These financial constraints are not echoed in the wordings of the OCP. When cancer treatment is dependent on an individual’s ability to pay for it, CCO cannot consider equity in cancer care without addressing this fundamental issue.

### End of Life Care

A goal of the OCP is to enhance the delivery of palliative care by bringing it closer to home. As such, the OCP commits to improve geographic access to palliative care. This objective however fails to contextualise the broader social circumstances of individuals that shape their end-of-life choices.

*“Patients will have discussions with their provider about advance care planning and will have the information they need to make informed decisions.”* ^6^

The main indicators of poor quality of end-of-life are: the number emergency department visits, and intensive care unit admissions within the last two weeks of life. Both of these have been consistently higher in cancer patients from the lowest income quintile in Ontario.^11^ Social identities such as gender, age, comorbidities intersect with the SDH such as income and education to predict end-of-life care.^37^ Indeed, studies demonstrate that up to 75 percent of cancer patients prefer to die at home.^38^ In Ontario however, only nine percent of cancer patients are able to do so, and this is primarily due to a lack of social support.^11^

Care at home requires caregivers. Studies have shown that caregivers who are younger, financially secure and physically fit are themselves optimally placed to provide end-of-life care at home.^37^ This is particularly apparent given that caregivers can lose up to 25% of their income as a result of caregiving responsibilities.^35^ However, to realise equitable end-of-life care, the OCP must take into account not only the psychosocial supports needed but also the financial leverages needed to enable this. The Quality Hospice Palliative Care Coalition of Ontario^39^ recommends: “expanded cash-for-care or direct payment funding schemes, and more flexible job benefits or protections for compassionate care leaves.” As direct advisor to the provincial government CCO is in a prime position to be making these specific policy recommendations but it fails to do so.

## Discussion

This paper explores inequities in cancer care in light of the “synergies of oppression” framework by McGibbon and McPherson.^22^ There is a growing body of evidence that links increased susceptibility to cancer, higher mortality, and poor quality of life with conditions of social disadvantage.^40–42^ Indeed, social identities/ geographies, and SDH intersect across every stage of the cancer care continuum^16^ such that individuals who are socially advantaged are able to optimally utilize the healthcare system, negotiate better care, and enhance their opportunities for survival.^20^

According to Whitehead,^26^ policies must at the very least acknowledge the existence of social inequities in health. One of the key goals for the OCP is equity; however, the OCP contextualises equity almost exclusively as an access-to-care issue and as a result most actionable targets and supports driven out of the plan intervene to overcome cultural and financial barriers to access. The role of social identities and intersections with the SDH across the cancer care continuum are not contextualised within the OCP. As a result, it falls short of acknowledging and making accommodations to cater to these population needs.

Innovations in cancer management imply that care is now increasingly delivered in the community or home. As such, individuals who lack social support are most likely to fall through the cracks during the delivery of care if their living and working conditions are not recognised. This is further compounded by rising levels of socioeconomic inequalities in Canada which increases the pool of socially disadvantaged individuals.

As a policy advisor to the government, the OCP represents a lost opportunity to link top-down biomedical care, with bottom-up reforms in social policy and planning that will facilitate the utilization of care by cancer patients. Furthermore, the OCP can promote “glocalization,” also described as local action towards health equity to mitigate the effect of global economic forces,^43^ by advocating for the appropriate allocation of social resources which will enhance the living and working conditions of cancer patients. This will enable socially disadvantaged patients to interact with the health system and benefit from universally-available healthcare.

One of the strengths of the OCP is the focus on Indigenous populations, and the Aboriginal (First Nations, Inuit, Métis or FNIM) Cancer strategy. A core emphasis of the FNIM plan is to build relationships and to nurture partnerships between Indigenous populations and CCO.^6^ It is well understood that this participatory approach will form the foundation of an equitable system where the cancer system can become accessible, acceptable and utilizable to the FNIM. This is reinforced by a data collection strategy to identify and measure equity gaps and create community cancer risk profiles.^6^

Despite the well-intentioned efforts of the FNIM health equity strategy of the current OCP, few considerations remain: (*i*) to prevent “othering” it will be important to shift the language of the plan and objectives from one of cultural relevance (where Indigenous cultures are respected, acknowledged and used to train and build programs) to one which is grounded in cultural safety^44^ (where the sociopolitical lived realities of generations of Indigenous populations have lead to the cancer inequities which we see today); (*ii*) there is a danger of clustering all vulnerable populations together. As such, the intrinsic heterogeneity that exists within population groups as a result of their social location may not be amenable to a single intervention and may inadvertently widen the health equity gap^45^; and (*iii*) data should be used not only to build evidence for local and community-based action, but data should also be used to advocate to the government for upstream policy action that will improve the distribution of the SDH.

As CCO prepares the next OCP, the OCP V, it must take into consideration its unique role as an interface between cancer patients and the government. Whilst a promise to deliver health equity to all Ontarians is noble, it must be backed by data, action and upstream political advocacy. To do this, CCO can assume governance roles for health equity that have been described by Labonte,^46^ such as : (*i*) The Watchdog: which monitors inequities in health and the SDH that underpin them; (*ii*) The Resource Broker: who ensures that adequate funding and resources are dedicated to tackling health inequities; (*iii*) The Community Developer: who works between health and community services to enhance the SDH; (*iv*) The Partnership Developer: who brings multiple stakeholders together to produce intersectoral action for health equity; and (*v*) the Advocate: who creates policy briefs to promote upstream action for health equity.

Inherent in this paper is the SDH approach, and analysis of the OCP IV was done with a strong bias towards finding evidence to support this theory. According to Hsieh,^23^ this is one of the greatest limitations of conducting a directed content analysis. However, the OCP is a strategic policy document with a clearly defined health equity goal. Furthermore, given that the OCP is one of few NCCPs across the globe to have a focus on health equity, this study presents an important analysis which is of relevance to all stakeholders in cancer systems as they seek to reduce inequities in health.

According to the 2030 Agenda for Sustainable Development it is a key healthcare imperative for nations to ensure that “no one is left behind” and that we are “reaching the furthest behind first.”^47^ As a result NCCPs are starting to build health equity goals and objectives into strategic priorities. As we move forward it will be necessary to collect more robust data on health equity. It will also be important to create indices that reflect the attainment of health equity goals, such as a percentage reduction in health outcomes between income groups, or improvement in healthcare utilization rates over time.^9^ However, it will be most significant to realise that health inequities are rooted in social inequities; and that the SDH play a profound role in cancer risk, outcomes and mortality. This will require NCCP’s to advocate for policies that influence the ability of patients to seek and benefit from care. Therefore NCCPs must not shy away from participating in political debates that subsequently influence social and health policy: As Navarro has succinctly put it, “you cannot speak of policies without touching on the politics.”^48^

## Conclusion

Health systems have a mandate to deliver quality health services. Increasingly quality indicators include health equity, and as a result health system policy plans are focused on strategies to reduce health inequities across the patient population whom they serve. The traditional role of health systems has been limited to the delivery and implementation of health services equally across the population. However, now that health systems are beginning to focus on health equity, it is important to realise that health inequities arise as a result of socially-based inequities. Therefore, the ability of patients to successfully receive care is dependent on their living and working conditions. Health system plans and strategic roadmaps must build this realisation into their policy narrative.

## Acknowledgements

I would like to thank the anonymous reviewers for their insightful feedback. I would also like to thank my mentor Dr. Dennis Raphael for his wisdom and guidance that have been crucial in the development of my work.

## Ethical issues

No human subjects were involved in the conduct of this study and therefore ethics approval was not required.

## Competing interests

Author declares that she has no competing interests.

## Author’s contribution

AS is the single author of the paper.

## Key messages

**Implications for policy makers**

- National Cancer Control Plans (NCCPs) are important tools to plan for strategic priorities in cancer control and care based on the social, demographic and economic needs of a defined population.
- Both the World Health Organization (WHO) and the United Nations place an emphasis on health equity so that the health divide between population groups based on social location can be reduced.
- Only a third of global NCCPs currently have a health equity focus, and even fewer NCCPs set tangible goals and metrics to evaluate how health equity will be measured and achieved.
- For cancer, the unequal distribution of the social determinants of health (SDH) creates an unequal field for the delivery of cancer care. These conditions profoundly influence cancer risk, outcomes and mortality.
- NCCPs that include health equity as a goal will need to recognise the living and working conditions of cancer patients that influence their health outcomes and be ready to advocate for the social policies that affect the utilization of healthcare.

**Implications for public**

Rising levels of income inequality are placing an increasing number of people in socially vulnerable circumstances. As a result, people who have access to fewer resources can have a hard time avoiding risk and maintaining good health. Indeed, when socially disadvantaged individuals they fall ill, it can be difficult for them to take time off work, and access medicines and care. Whilst many healthcare providers are aware of health inequities, the goals and objectives of health systems fail to encompass consideration of forces beyond the delivery of healthcare that can influence health outcomes. Health systems must be responsive to the living and working needs of the patients who they serve. And where needed health systems must raise a political voice to protect patients and promote their ability to seek and benefit from healthcare.
